# Enhanced vapor transport in membrane distillation via functionalized carbon nanotubes anchored into electrospun nanofibres

**DOI:** 10.1038/srep41562

**Published:** 2017-01-30

**Authors:** Alicia Kyoungjin An, Eui-Jong Lee, Jiaxin Guo, Sanghyun Jeong, Jung-Gil Lee, Noreddine Ghaffour

**Affiliations:** 1 School of Energy and Environment, City University of Hong Kong, Tat Chee Avenue, Kowloon, Hong Kong, China; 2 King Abdullah University of Science and Technology (KAUST), Water Desalination and Reuse Center (WDRC), Biological and Environmental Science & Engineering (BESE), Thuwal 23955-6900, Saudi Arabia

## Abstract

To ascertain membrane distillation (MD) as an emerging desalination technology to meet the global water challenge, development of membranes with ideal material properties is crucial. Functionalized carbon nanotubes (CNTs) were anchored to nanofibres of electrospun membranes. Covalent modification and fluorination of CNTs improved their dispersibility and interfacial interaction with the polymer membrane, resulting in well-aligned CNTs inside crystalline fibres with superhydrophobicity. Consideration for the chemical/physical properties of the CNT composite membranes and calculation of their theoretical fluxes revealed the mechanism of MD: CNTs facilitated the repulsive force for Knudsen and molecular diffusions, reduced the boundary-layer effect in viscous flow, and assisted surface diffusion, allowing for fast vapor transport with anti-wetting. This study shows that the role of CNTs and an optimal composite ratio can be used to reduce the gap between theoretical and experimental approaches to desalination.

Membrane distillation (MD), a thermally driven water-purification process, is emerging as a viable option for water treatment. In particular, MD is promising for seawater distillation due to its high energy efficiency and high water recovery compared to other major technologies, such as multi-stage flash and multiple-effect distillation^1^,^2^,^3^. MD heavily relies on the material properties of the membrane to determine its efficiency. For water treatment, membrane that achieves high permeability without wetting (i.e., hydrophobic), has appropriate pore size (but with high rejection) with narrow pore-size distribution, and has a tortuosity close to one (cylindrical) is desired^4^,^5^. For this reason, nanocomposite membranes have recently been gaining credit for their high functionality and selectivity^6^,^7^,^8^,^9^,^10^,^11^. In particular, carbon nanotubes (CNTs) are a promising nanomaterial for membrane fabrication because they can modify the membrane’s architecture to achieve superior physical/chemical properties. Efforts to introduce CNTs into membranes have been focused on their potential application to create effective, efficient materials that can be used to reduce environmental impact and protect water resources^12^,^13^,^14^,^15^.

To employ any type of nanomaterial in polymer nanocomposites, adequate dispersion and appropriate interfacial adhesion between the nanomaterials and the polymer matrix have to be assured^16^. Although previous studies have suggested that CNTs promote the transport of water vapor and gas across membranes^17^,^18^, optimally exploiting the intrinsic properties of CNTs remains challenging^19^. Hence, efforts to develop dispersion and functionalization techniques that optimize carbon nanocomposites are necessary^20^ before the widespread commercialization of nanocomposites with economic significance can be realized^19^. For example, difficulties in dispersing CNTs has limited the application of nanocomposite membranes to water treatment^21^.

Here, we demonstrate the synthesis of a functionalized CNT-composite electrospun membrane for practical applications to MD for desalination. Inspired by previous researchers, we develop a scale-able, well-ordered membrane using multi-walled CNTs (MWCNTs) by focusing on their dispersion and interaction with polymer materials. We introduce an easy, versatile technique for building a three-dimensional (3D) superhydrophobic CNT-poly (vinylidene fluoride-hexafluoropropylene) (PVDF-HFP) composite membrane by electrospinning. To optimize the performance of the membrane for desalination, we incorporate different concentrations of CNTs (~3%) into the PVDF-HFP polymer and the resultant membranes are tested in direct contact MD (DCMD). Finally, we quantify the degree of CNT functionalization and dispersion into the nanocomposites by comparing experimental data with theoretically simulated data, and we discuss the possible mechanism of vapor transport in the pores of optimized CNT incorporated membranes.

## Results

### Dispersion of CNTs

**Figure 1:**
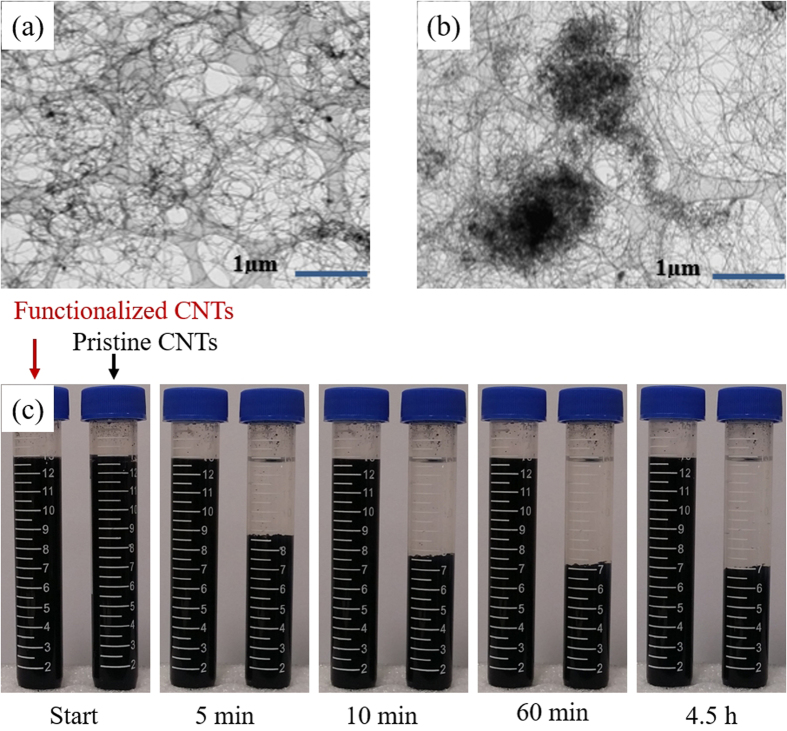
TEM images of (**a**) well-dispersed functionalized CNTs and (**b**) aggregated pristine CNTs. (**c**) Suspension stability of functionalized and pristine CNTs.

Covalent modification to CNTs improved their dispersion in the solvent and produced strong interfacial bonds with polymers, allowing the CNT nanocomposites to possess multifunctional properties^21^. TEM images show that the dispersion of the CNTs improved after the functionalization (Fig. 1a) compared to the original pristine CNTs (Fig. 1b). The pristine CNTs showed severe agglomeration in entangled bundles, whereas the agglomeration was significantly reduced after functionalization. Moreover, the functionalization rendered greatly improved stability to the suspension of CNTs in DMF/acetone (4:1 wt%), as shown in Fig. 1c. The pristine CNTs immediately began to settle after 1 h of sonication and 1 min of vortex mixing, while functionalized CNTs remained well suspended for about 24 h, far exceeding the 4.5-h electrospinning process.

### Interfacial interaction between CNTs and PVDF-HFP polymer

**Figure 2:**
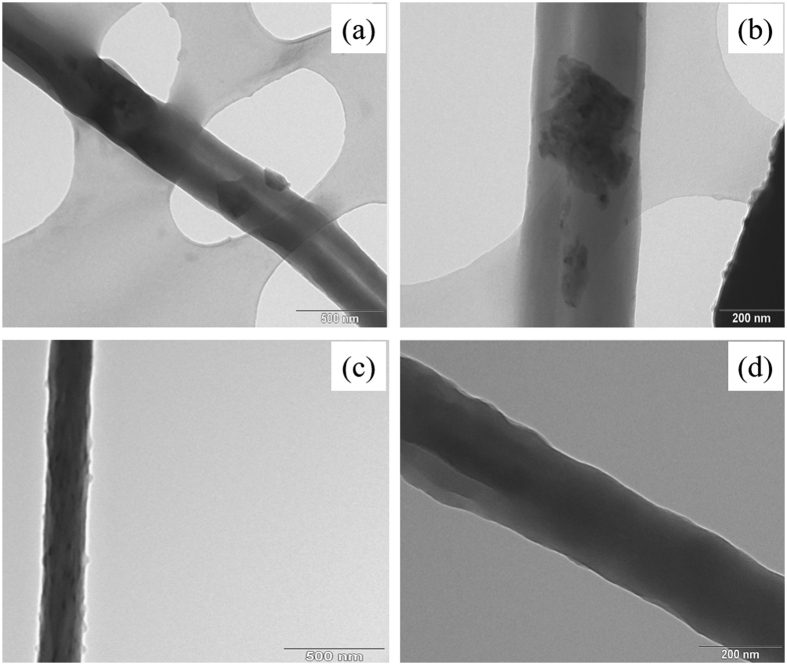
TEM images of (**a**) nanofibres composited with pristine CNTs (×20,000) and (**b**) (×50,000), and (**c**) nanofibres composited with functionalized CNTs (×20,000) and (**d**) (×50,000).

Agglomeration between pristine CNTs in the electrospun nanofibres was strong due to the inter-sp^2^-bonded carbon atoms (also known as π-π interactions) rather than due to the carbon-polymer interactions^22^. The TEM images in Fig. 2a and b show scraps or fragments of pristine CNTs on the fibres caused by poor dispersion during dope-solution preparation and inadequate interfacial interactions with the PVDF-HFP polymer during fibre formation. Figure 2c and d demonstrate the good alignment of functionalized CNT nanocomposites on the nanofibres. Surface fluorosilanization using 1 *H*,1 *H*,2 *H*,2*H*-perfluorooctltriethoxysilane (FTES) reduced the number of hydroxyl (OH) and hydrogen (H) groups on the functionalized CNTs-COOH while FTES underwent hydrolysis and condensation, which increased hydrophobicity and allowed individual or disentangled CNTs to embed nicely inside the polymer matrix. Furthermore, the salinized open edges and defect in the sidewalls of the CNTs became chemical anchors for additional adhesion to the polymer either by hydrogen boding or partially polar covalent bonding with polymer. Positioning of highly electronegative fluorine (F) at the end of the bond created a partially negative charge that led to shared electrons bonding, polar covalent bonding. This improved CNT-polymer bonding stabilized the well-dispersed functionalized CNTs in the polymer-dope solution during the electrospinning process and hindered secondary agglomerations by the CNTs while the fibre was being formed. These results are in accordance with those of a previous report^23^.

### CNT alignment in the nanofibres during membrane fabrication

**Figure 3:**
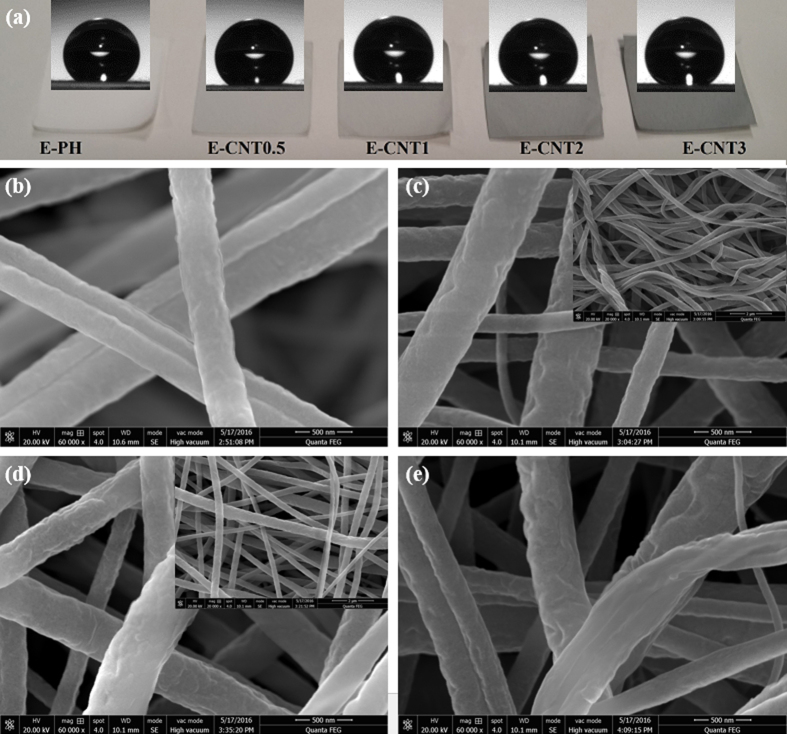
(**a**) Pictures of E-PH and E-CNTs membranes with contact angles and (**b–e**) FE-SEM images of E-PH, E-CNT1, E-CNT2, and E-CNT3 membranes (×60,000 and the inset images are ×20,000 magnification).

Figure 3a shows pictures of the electrospun 20 wt% PVDF-HFP without CNT (hereinafter, E-PH) and electrospun PVDF-HFP composite fabricated by electrospinning using 0.5, 1, 2 or 3 wt% CNTs (hereinafter, E-CNT0.5, E-CNT1, E-CNT2 and E-CNT3) with their contact angles. Figure 3b–e show their surface morphologies as observed by field-emission scanning electron microscopy (FE-SEM). The E-CNT membranes exhibit more crystalline surfaces than the E-PH membranes, caused by nano-scaled protrusions of CNTs, increasing their roughness. In addition, FE-SEM shows reinforced nanofibres with orderly aligned nanocrystalline CNTs structures along the fibre.

To closely examine the roughness of a large sample area (88.8 μm × 66.4 μm) of precision surfaces in 3D, optical profile images were obtained and presented as Supplementary Fig. S1. The E-CNTs (R_a_: 12.04~2.53 μm) membranes exhibit a rougher surface than that of the E- PH membrane (R_a_: 1.35 μm). In addition, the images show that higher concentrations of CNTs lead to increased roughness. The colors corresponding to the surface height were visualized in the order of red > green > blue.

To date, many researchers have attempted to enhance the water resistance of the membrane surface by aligning nanomaterials on the fibre surface so as to increase the surface roughness of membrane and form numerous air pockets^20^,^24^. The E-CNT membranes fabricated in this study had CNTs positioned in the interior of fibres as if they were fillers to engineer wrinkling in the fibres, as can be seen in the TEM images (Fig. 2c and d). The compressive hoop stress from the rapid evaporation of the solvent can create unique wrinkled-patterned surface topographies^25^ and the relatively slower diffusion rate of molecules compared to the shrinking speed of the droplet can form wrinkled polymeric microparticles^26^. In other words, if the polymer fibres are unable to congregate well during the formation of polymeric particles or fibres, wrinkled patterns can occur as the evaporation of the solvent shrinks the polymer fibres. As such, the severely wrinkled patterns found in the CNTs embedded in fibres are likely due to the interfacial behavior between the polymer and the CNTs, where the CNTs hindered the polymer molecules from diffusing back into the fibre or retaining sufficient entanglements to form uniformly distributed polymer chains during solvent evaporation. In addition, little distinction was found in the severity of the wrinkled patterns among the E-CNTs membranes, which implies that the different concentrations (0.5 to 3 wt%) of CNTs had only a minor affect on the ability of polymer molecules to congregate toward the fibre core.

**Figure 4:**
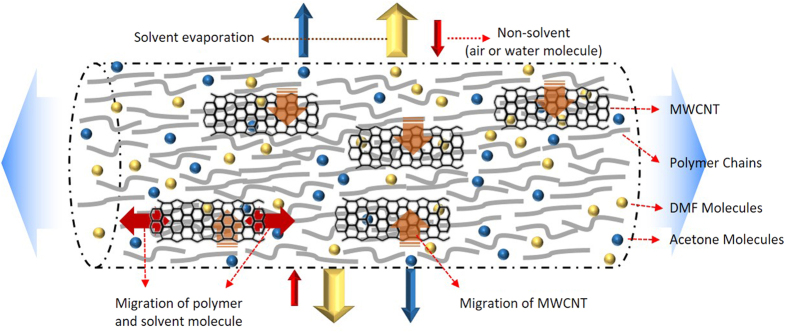
A schematic description of the migration of molecules during solidification of CNT-embedded polymer nanofibres.

The possible explanation for the movement of CNTs into the fibre core is illustrated in a schematic diagram (Fig. 4). Unlike other nano-fillers (e.g., TiO_2_, SiO_2_, and Al_2_O_3_), CNTs have a unique hollow cylindrical structure. When CNTs are dispersed in the dope solution, they become filled with the dissolved polymer. During the electrospinning process, the polymer fibres shrink due to the evaporation of solvents, and stretch longitudinally due to repulsive forces as they travel from the nozzle to the collector. On the fibre surface, the solvent molecules migrate to the shell and evaporate into the air and the polymer molecules congregate while moving to the core. Meanwhile, the CNTs filled with polymer solution are not likely to separate from the polymer, and thus, move to the core like polymer molecules. This explains why the CNTs were not observed in the FE-SEM images in Fig. 3. At the same time, the open-ended structure of the CNTs allows the longitudinal movement of solvent or polymer molecules inside of them, resulting in a well-aligned CNT configuration, as observed in the TEM image (Fig. 2c and d) as well as the schematic description in Fig. 4.

### Hydrophobicity of E-CNT electrospun membranes

The images obtained from the goniometry test on the membranes and their corresponding water contact angles are presented in Table 1. The contact angle of the C-PVDF and the E-PH membrane were 123 ± 1.9° and 142.4 ± 1.7°, respectively. The contact angle of E-CNT membranes increased with increasing CNT concentration (E-CNT0.5 = 146.0 ± 0.9° and E-CNT2 and E-CNT3 > 150°), indicating greater hydrophobicity than E-PH membranes. Thus, functionalizing the membranes to increase their surface roughness increased water contact angles and hydrophobicity of the membrane.

### Nanofibre diameter, pore-size distribution and liquid-entry pressure

**Figure 5:**
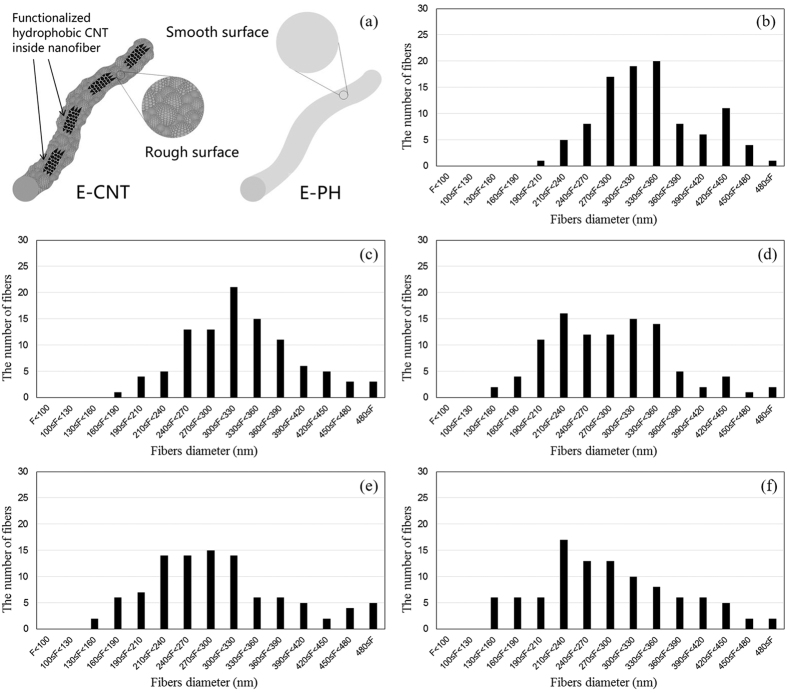
Schematic diagram of PH and CNT fibres (**a**) and fibre dimeter distribution in (**b**) E-PH, (**c**) E-CNT0.5, (**d**) E-CNT1, (**e**) E-CNT2, and (**f**) E-CNT3.

The SEM images also showed changes in the nanofibre dimensions after functionalized CNTs were incorporated into the membrane. The nanofibre diameters of the E-CNTs membranes were generally smaller than that of the E-PH. The average and distribution of nanofibre diameters for each electrospun membrane are presented in Table 1 and Fig. 5, respectively. The nanofibres of the E-PH membrane were the thickest with an average fibre diameter of 336.1 ± 65.1 nm. The E-CNT0.5 and E-CNT1 membranes possessed nanofibres with an average diameter of 324.9 ± 72.0 and 286.1 ± 74.9 nm, respectively. Results showed that nanofibre diameter becomes thinner at higher concentrations of CNTs; a similar result was reported by Liu *et al*.^27^.

The viscosity of the polymer solution was correlated to the size of the nanofibre’s diameter. Adding nanomaterial to the polymer solution increases the solution’s viscosity and causes greater entanglement, thereby increasing nanofibre diameter^28^. Meanwhile, adding CNTs to the polymer solution strengthens the repulsive force because CNTs have high electrical conductivity, decreasing the diameter of the nanofibres. Therefore, the diameters of nanofibres in the E-CNTs were determined by the balance between increased viscosity and increased repulsive force. As shown in Fig. 5, compared to the nanofibre-diameter distribution of the E-PH membrane, there were relatively fewer thick nanofibres (over 420 nm) in the E-CNT0.5 membrane. When the concentrations of CNTs increased (E-CNT1 to E-CNT3), the ratio of thin nanofibres increased, and the number of middle-sized nanofibres decreased although their average nanofibre diameters were similar.

The pore-size distributions of the membranes fabricated in this study were narrower and uniform despite their relatively larger average pore sizes. During MD, a narrow pore-size distribution is essential to prevent wetting (or partial wetting)^29^. LEP was measured to quantify the long-term stability of the membranes for MD application and their values are summarized in Table 1. The LEP for the E-PH, E-CNT0.5 and E-CNT1 membranes were 79, 68, and, 69, respectively. CNT concentrations greater than 2 wt% led to poor LEP in accordance with the Laplace equation (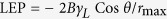), which defines LEP to be directly proportional to the cosine of the water contact angle and inversely proportional to the largest pore size. Although the water contact angle was improved after incorporating functionalized CNTs into the membrane, the maximum pore size increased and slightly lowered LEP. As previously mentioned, the pore size of the E-CNT3 membrane was the largest, as the instability from the extremely high and unequal charge density and the re-agglomeration of the CNTs led to abnormal bead structure on the nanofibres. Since MD is not a pressure-driven process, the LEP values of all membranes fabricated in this study were reasonably suitable for MD operation, however, low LEP due to the increase in pore size may reduce the long-term stability of the membranes.

### MD performance

**Figure 6:**
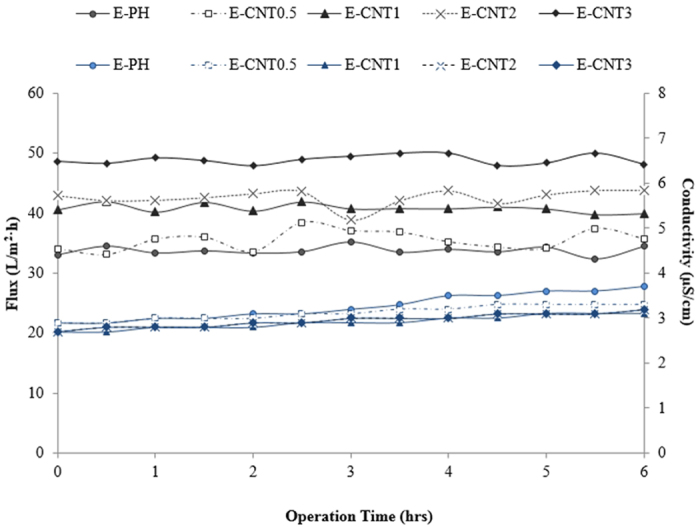
DCMD fluxes treated with 35 g/L of NaCl solution using the E-PH and the E-CNTs membranes embedded with different concentrations of CNTs at a feed temperature of 60 °C.

Flat-sheet DCMD tests were performed with the E-PH and E-CNTs membranes, and their water flux was relatively stable throughout the 6 h of operation as presented in Fig. 6. The largest average water flux value was measured for the E-CNT3 membrane at 48.1 L/m^2^∙h, which was about 60% higher than that of the C-PVDF membrane (20.2 L/m^2^∙h) and 35% higher than that of the E-PH membrane (33.6 L/m^2^∙h). The suitability of a membrane for use in MD is determined by several properties. Supplementary Table S1 lists the MD performance of various electrospun membranes with improved hydrophobicity. Although electrospun membranes are inherently favourable for enhancing permeability due to its interconnected porous structure, membrane thickness causes a large resistance against vapor movement across membranes resulting in low flux^28^. Permeability may also be increased with large pore size, which can be effective as long as membrane hydrophobicity is high enough to retard pore wetting efficiently^30^,^31^. Including CNTs in the polymeric solution achieved a hydrophobic surface and MD performance with a relatively small amount of nanofillers compared to other nanocomposite membranes^32^,^33^. At the highest concentration of CNTs (E-CNT3), prominent permeability was achieved without severe wetting due to the large pore size in the membrane, the thin membrane layer of 88 μm, and sufficient hydrophobicity. When the feed salinity of DCMD was increased from 0 to 70 g/L NaCl, the permeate flux gradually lowered in both E-PH and E-CNTs membranes. However, the flux dropped because the salinity of the E-CNTs was lower than that of the E-PH, indicating that the CNT composites are less susceptible to salinity and that the E-CNTs had less concentration polarization effect on their membrane surfaces.

The rejection efficiencies of all membranes were superior (above 99.98%) and the E-CNTs showed lower permeate conductivity, which indicates higher permeate quality, while the E-PH exhibited slightly higher permeate conductivity despite the fact that the flux was lower. Therefore, the E-CNT membranes fabricated in this study are suitable for seawater desalination in MD operations, and more importantly, are an innovative solution to environmental challenges in water treatment.

## Discussion

### Effect of CNTs on E-CNT membranes

To date, the functionalization of CNTs and their dispersion in nanocomposites have been studied using qualitative methods, such as images. Quantitative methods are necessary to establish the effects of incorporating CNTs in nanocomposites and to determine optimal concentrations of CNTs for specific applications. Thus, we compared the results from the quantitative methods employed here with simulated data to evaluate the effect of CNTs on the flux of E-CNT membranes and to identify the mechanism of mass transfer in MD. Simulated water flux was calculated, based on a previously developed model^34^, by multiplying the mass transfer coefficient (*C*) by the difference in water vapor pressure (*Δp*_*f,p*_):


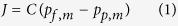


where *p*_*f,m*_ and *p*_*p,m*_ are the water vapor pressure of feed and permeate sides, respectively.

The dusty gas model, which consist of the three models, Knudsen diffusion, molecular diffusion, and Poiseuille or viscous flow transition, was applied to describe the water flux through the membrane as follows:


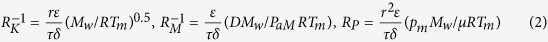



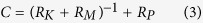


where *R*_*K*_, *R*_*M*_, and *R*_*P*_ represent Knudsen diffusion, molecular diffusion, and viscous flow, respectively; *p*_*m*_ represents water vapor pressure inside the membrane (*p*_*m*_ = (*p*_*f,m*_* + p*_*p,m*_) / 2); and *P*_*aM*_ is the log-mean air pressure at both sides of the membrane. The binary diffusion coefficient (*D*) is calculated using the Fuller-Schettler-Giddings empirical equation, which can be expressed as below:


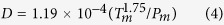


Calculations of bulk temperatures, velocities and pressures took into account the grid distribution node in the module length as well as the transmembrane temperatures, T_m_ at both feed and permeate sides. Water vapor pressures at the feed and permeate sides (*P*_*f,m*_ and *P*_*p,m*_ (≈*P*_*w,m*_)) can be determined by the solution’s molar fraction. For a dilute aqueous solution of salts, the following approximation is often considered^34^,^35^:


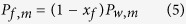


where *x*_*f*_ is the molar fraction of non-volatile solute and *P*_*w,m*_ is the pure water vapor pressure. *P*_*w,m*_ can be evaluated with the Antoine equation as follows:


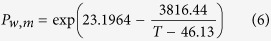


**Figure 7:**
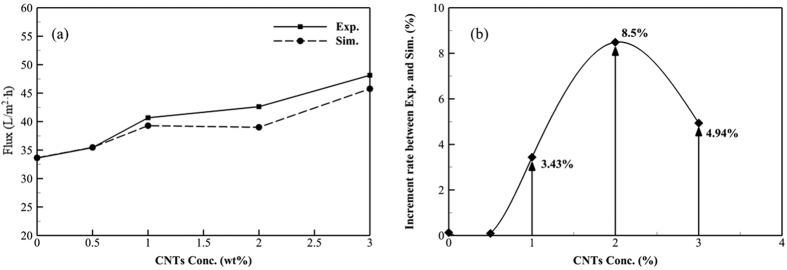
(**a**) Effect of the changed physico-chemical properties, such as mean pore size, porosity, thickness, and thermal conductivity, on the experimentally measured (Exp.) and simulated (Sim.) water flux in DCMD using different concentrations of CNTs (wt%) embedded in E-CNT membranes; (**b**) the difference between experimental and simulation fluxes with respect to the concentration of CNTs (wt%).

The experimental study used a small-scale experimental set-up, which was well insulated during the experimentation to ensure negligible heat loss from module to ambient. Comparison of the experimental data and simulated data clearly show that increasing the concentration of CNTs improves the average water flux, albeit with some discrepancy (Fig. 7a). The difference in water flux between the experimental and the simulated data with respect to the concentration of CNTs is plotted in Fig. 7b.

Figure 7a shows the effect of the changes in physico-chemical properties, such as mean pore size, porosity, thickness, and thermal conductivity, on the measured (Exp.) and predicted (Sim.) water flux with respect to the concentration of CNTs (wt%). In the DCMD operation with 35 g/L NaCl at 60 °C feed temperature, the measured water flux increased from 33.6 to 48.1 L/m^2^∙h as the concentration of CNTs was increased from 0 to 3 wt% while the simulated water flux increased from 33.6 to 45.7 L/m^2^∙h. Thus, low concentrations of CNTs (0 and 0.5 wt%) were insufficient to increase the permeate flux more than the theoretical value while higher concentrations of CNTs (1, 2 and 3 wt%) were sufficient to increase the water flux above simulated conditions.

Alternatively, Fig. 7b shows higher experimental (Exp.) water flux than simulated (Sim.) flux for intermediate concentrations of CNTs (0.5 to 2 wt %) and lower flux rates at the highest concentration of CNTs (3 wt %). This implies that the E-CNT3 was not fabricated with well-dispersed CNTs. Since the E-CNT0.5 membrane did not exhibit enhanced water flux compared to the theoretical value, for the reasons stated above, it can be concluded that the E-CNT2 membrane, which had 8.5% better water flux than that of the simulated water flux, was the best membrane for DCMD. Changes in physio-chemical properties of the membrane (pore size, thickness, porosity, and thermal conductivity) by the incorporation of CNTs directly affected the permeate flux, especially in terms of mass transfer. Ultimately, these properties are influenced by indirect factors, such as LEP, hydrophobicity, fibre diameter, and dispersibility of CNTs. In summary, mass transfer can be increased by altering and enhancing geometric factors, such as increased pore size and porosity, with the incorporation of well-dispersed functionalized CNTs.

### Possible mechanism of a 3D superhydrophobic E-CNT membrane

**Figure 8:**
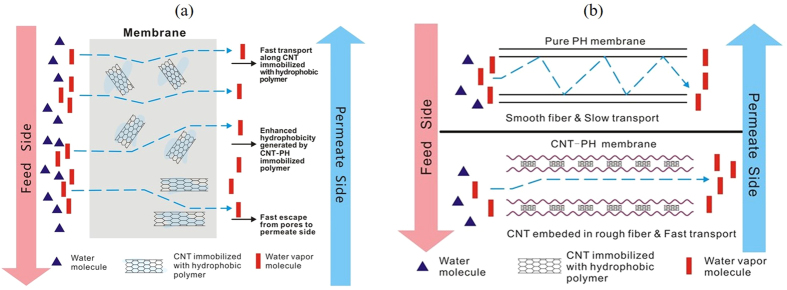
(**a**) Possible mechanisms of enhanced vapor transport in the E-CNT membranes. (**b**) Effect of increased roughness on vapor transport in the E-CNT membranes.

The superhydrophobicity of even the inner pores as well-dispersed CNTs in the E-CNT membranes that resulted in the homogeneous embedding fabricated in this study sets them apart from other superhydrophobic 3D membranes fabricated by electrospinning. As a result, the 3D superhydrophobic E-CNT membranes fabricated in this study achieved superior mass transfer through the pores of the membrane through the mechanism summarized in Fig. 8a.

The superhydrophobic pore walls repel water vapor molecules thereby mitigating negative effects, such as friction or adsorption from occurring between the pore wall and the water vapor molecule. This assessment is in line with that dictated by Knudsen and molecular diffusions on mass transfer resistance. Along this line of logic, resistance of mass transfer can also be decreased by reducing the boundary-layer effect related to viscous flow. In other words, the repulsion force provided by superhydrophobicity can reduce the non-slip condition zone near the pore wall^36^. Therefore, incorporating CNTs can lead to faster vapor transport through the membrane than in the absence of CNTs. The superhydrophobicity endowed by CNTs also increases the repulsive energy of the pore and vapors, which increases Knudsen and molecular diffusion coefficients, and reduces the boundary-layer effect, which accelerates viscous flow in the pores of the membrane. In other words, the mass-transfer behavior within the E-CNT membrane can be described as having shorter wall collision distance and less molecule collision to facilitate the flow of vapors through the membrane by decreasing loss of friction. Subsequently the tendency of the pores to become wet with liquid decreases, such that more transport of pure vapor can occur^37^ because it moves through the pores like a magnetic levitation train.

Transport of vapor transport may also be enhanced by the agglomeration of CNTs in the nanofibres, which causes the formation of beads on the surface, due to their strong Van der Waals attraction^38^, increasing the overall surface roughness of the membrane. We predict that the additionally formed hierarchical structures with nano- and micro-roughness can reduce the formation of boundary layers promoting a high driving force in the E-CNT membrane. Figure 8b illustrates our proposed mechanism for how the incorporation of CNTs changes the inner path and the pore shape or geometry in the E-CNT membrane, increasing the surface roughness, to facilitate vapor transport. Theoretical fluxes calculated in consideration of chemical/physical properties of the membranes unfolded the MD mechanism using CNTs composite membranes. It was revealed that CNTs facilitated the repulsion force for Knudsen and molecular diffusions, reduced the boundary layer effect in viscous flow, and assisted surface diffusion, allowing the fast vapor transport with anti-wetting. This study will reduce the gap between experimental desalination and theoretical one with the studied role of CNTs and their optimal composite ratio for CNTs composite membrane.

## Methods

### Materials

Three types of membranes were used for MD in this study: commercial, electrospun without CNT incorporated, and electrospun with CNT incorporated. The commercial membrane was a 0.45-μm PVDF-HFP membrane, named C-PVDF (Durapore^®^ Membrane Filters, Merck Millipore Ltd), which was used as the reference membrane to test for MD performance. The electrospun membrane without CNT incorporated was a 20 wt% polyvinylidene fluoride-hexafluoropropylene (PVDF-HFP) membrane (E-PH). Composite CNT-PH membranes were fabricated by electrospinning using, 0.5, 1, 2 or 3 wt% CNTs to PH (E-CNT0.5, E-CNT1, E-CNT2 and E-CNT3). Commercial pristine MWCNTs (purity > 90 wt% and bulk density = 0.04~0.08 g/cm^3^) were obtained from Carbon Nano-material Technology Co., Ltd., Korea with 5~20 nm in diameter and ~10 μm in length. Their specific surface area was 155 ± 5 m^2^/g.

### CNT dispersion and functionalization

As stated earlier, effective incorporation of the CNTs to the polymer requires i) proper dispersion of the CNTs and ii) appropriate interfacial adhesion between the CNTs and the polymeric matrix. Because pristine MWCNTs are supplied in the form of entangled bundles with extremely large surface areas they are intrinsically difficult to disperse. Hence, to adequately disperse the CNTs and achieve appropriate interfacial adhesion between CNTs and the polymer matrix, after ultrasonication, the CNTs were functionalized with polar covalent bonds by surface flurosalinization, increasing their hydrophobicity. These functionalization processes appeared to cause little damage to the CNTs, while facilitating the fabrication of a well-dispersed membrane by inhibiting the re-agglomeration of CNTs and producing interfacial bonds with the polymer.

### Covalent functionalization of CNTs

Covalent functionalization chemically produces defect sites on the CNTs^21^ that enable strong interfacial bonds with the polymer, endowing beneficial functional properties. For oxidization, 2 g of the ultrasonicated CNTs were mixed with 400 mL of concentrated nitric acid under a reflux for 18 h. The oxidization process creates defect sites at the open ends of the CNTs, which stabilize by bonding with the carboxylic acid (–COOH) group to facilitate further chemical reactions (see Supplementary Fig. S2). After oxidization, the CNTs were centrifuged, rinsed with deionized (DI) water, air-dried overnight, and then added to 50 mL toluene and sonicated for 1 h to achieve uniform dispersion of the CNTs.

### Surface fluorosalinization of CNTs

The CNTs were further functionalized with FTES to endow hydrophobicity and improve the dispersion of CNT particles^39^. The low surface free energy of FTES allows stable carbon-fluorine (C-F) bonds to form on the surface of CNTs^40^. Moreover, hydrolysis of FTES causes a silane-based film of FTES to form on the surface of CNTs, conveying additional hydrophobicity also known as surface fluorosilanization^41^. The FTES solution was prepared by mixing 0.5 g of FTES with 0.75 mL of DI water and 50 mL of toluene for 1 h. The solution was then added dropwise to the CNTs in the toluene solution and the mixture was continually stirred for 18 h in a glove box. The CNTs were collected through centrifugation and dried at 120 °C for 3 h and then heated up to 180 °C for 30 min to completely remove any unreacted FTES.

### Fabrication of 3D electrospun membranes

The main dope solution for the electrospun membrane was prepared by mixing PH (*M*_*w*_ = 455,000 g/mol) (polymer) with lithium chloride (LiCl) (0.005 wt%) (additive) and *N*-dimethylformamide (DMF) and acetone (solvents) in a composition ratio of PH:DMF:acetone = 20:64:16. All the chemicals used in this study were purchased from Sigma-Aldrich. The dope solution was electrospun by ejecting onto a rotating collector 15 cm away from the tip of the nozzle at a rate of 0.7 mL/h (see Supplementary Fig. S3). A positive voltage of 18 kV was applied across the tip-to-collector distance.

The E-PH membrane was fabricated using 20 wt% PH with only the main dope solution. The E-CNTs membranes were fabricated by mixing different concentrations (0.5, 1, 2 and 3 wt% to PH) of functionalized CNTs with the main (PH) dope solution prepared previously. The compositions of dope solutions for membrane fabrication can be found as Supplementary Table S2 and elsewhere^42^. Electrospinning and post-treatment processes of the E-CNT membranes were the same as that used to fabricate the E-PH membrane, with the exception of the voltage applied across the tip-to-collector (i.e., 19 kV for the E-CNTs membrane). Membranes were placed in a fume cupboard 60 °C for overnight to eliminate any residual solvent.

### Membrane characterization

The surface morphology of the membranes was observed by FE-SEM (Quantum 450 FEG, FEI, USA) and transmission electron microscopy (Philips CM20 TEM). Surface topography was measured by an optical profiler for accurate 3D surface height measurements of precision surfaces (Wyko NT9300, Vecco, USA). A capillary flow porometer (Porometer, POROLUX™ 1000, Germany) was used to measure the pore size, pore-size distribution, and liquid entry pressure (LEP) of the membranes. A tensile test was conducted to measure the mechanical properties of the membranes using tensile strength measurements (Lloyd-Ametek LS1 material testing machine, USA). The water CONTACT ANGLE was measured by a contact angle meter (KRUESS GmbH DSA25S), and the thermal conductivity of the membranes was obtained using a thermal conductivity analyzer (C-Therm TCi, C-Therm Technologies, Canada). More detailed descriptions of characterization methods can be found elsewhere^42^.

### Membrane distillation operation

The DCMD experimental setup used in this study is presented in Supplement Fig. S4. The configuration of the membrane flow channel was 6.1 cm × 1.6 cm with an effective membrane surface area of 9.76 cm^2^. Three different feed solutions (0, 35, and 70 g/L of NaCl) were heated to 60 °C and the permeate solution (DI water) was maintained at 20 °C. Both solutions were circulated through both sides of the flat sheet membrane cell at a rate of 400 mL/min for 6 h in a counter-flow manner. The permeate flux was recorded in real-time with a data-logging system connected to a balance in the permeate side.

## Additional Information

**How to cite this article**: An, A. K. *et al*. Enhanced vapor transport in membrane distillation via functionalized carbon nanotubes anchored into electrospun nanofibres. *Sci. Rep.* **7**, 41562; doi: 10.1038/srep41562 (2017).

**Publisher's note:** Springer Nature remains neutral with regard to jurisdictional claims in published maps and institutional affiliations.

## Supplementary Material

Supplementary Information

## Figures and Tables

**Table 1:** Characteristics of the electrospun membranes fabricated in this study.

Membrane	Mean pore size (μm)	Maximum pore size (μm)	Thickness (μm)	Porosity (%)	LEP (kPa)	Contact angle (Degree)	Thermal conductivity (W/mK)	Nanofibre diameter (nm)
C-PVDF	0.450	0.550	100 ± 1	72.0	117.2 ± 0.5	123.0 ± 1.9	—	—
E-PH	0.590	0.621	88 ± 8	89.3	79.7 ± 0.8	142.4 ± 1.7	0.052	336.1 ± 65.1
E-CNT0.5	0.647	0.716	93 ± 3	89.4	68.1 ± 7.1	146.2 ± 1.2	0.055	324.9 ± 72.0
E-CNT1	0.758	0.927	88 ± 3	89.8	69.0 ± 2.1	148.2 ± 2.1	0.061	286.1 ± 74.9
E-CNT2	0.755	1.040	85 ± 2	90.3	55.1 ± 2.2	150.6 ± 1.3	0.063	285.6 ± 82.0
E-CNT3	1.120	1.680	88 ± 3	89.4	40.5 ± 2.8	150.4 ± 0.8	0.065	287.7 ± 89.9
