# Supplementary material for: Enhanced vapor transport in membrane distillation via functionalized carbon nanotubes anchored into electrospun nanofibres
Source: Sci Rep. 2017 Jan 30;7:41562. doi: 10.1038/srep41562 (PMC5278503; doi:10.1038/srep41562)
Supplement: Supplementary Information [file srep41562-s1.pdf]

Enhanced vapor transport in membrane distillation via functionalized carbon nanotube anchored into electrospun nanofibers

Alicia Kyoungjin AN[1*], Eui-Jong Lee[1], Jiaxin Guo[1], Sanghyun Jeong[2], Jung-Gil Lee[2], and Noreddine Ghaffour[2]

[1]School of Energy and Environment, City University of Hong Kong, Tat Chee Avenue, Kowloon, Hong Kong, China,
[2] King Abdullah University of Science and Technology (KAUST), Water Desalination and Reuse Center (WDRC), Biological and Environmental Science & Engineering (BESE), Thuwal 23955-6900, Saudi Arabia
**Corresponding author's e-mail: alicia.kjan@cityu.edu.hk*

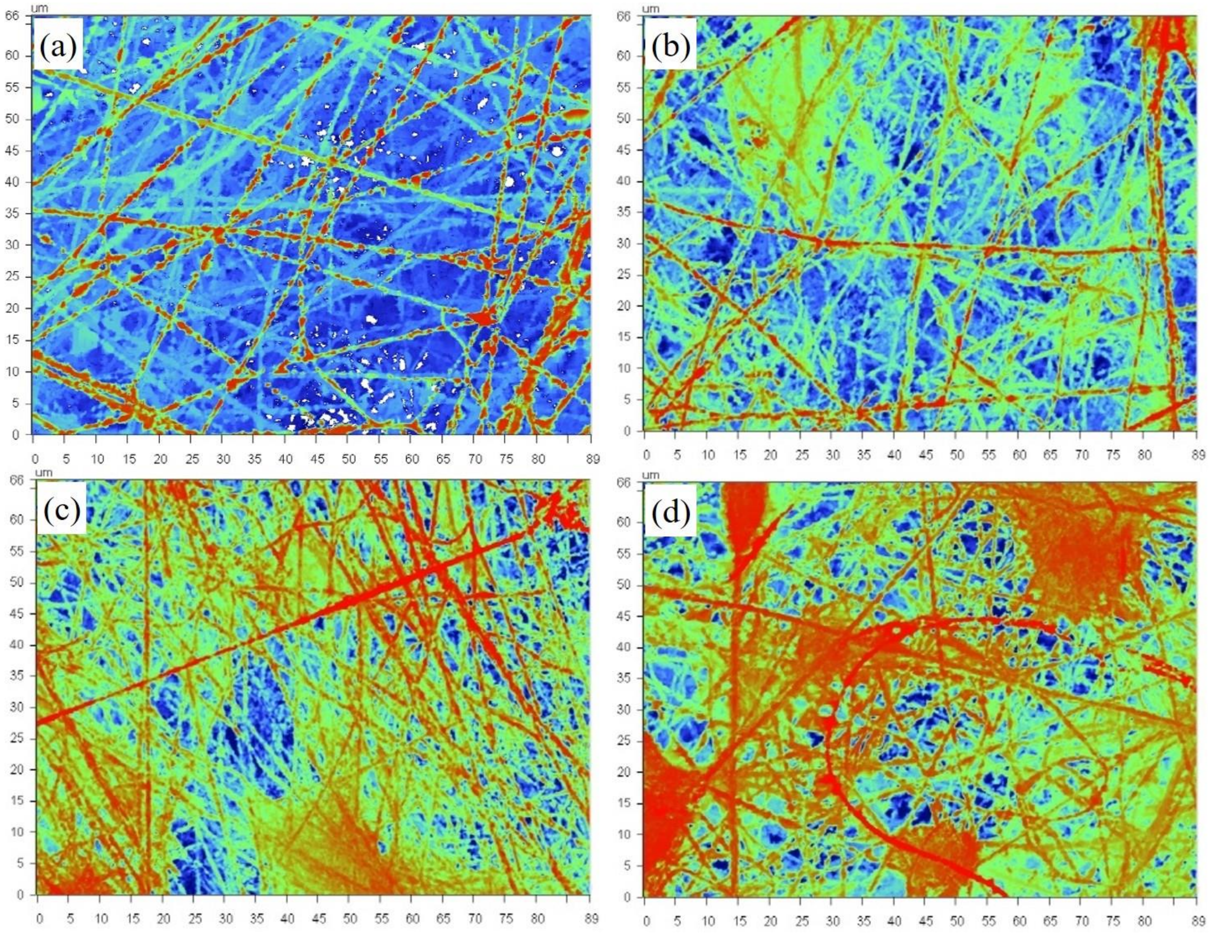

Supplementary Figure S1. Optical profiler images (88.8 μm × 66.4 μm) of (a) E-PH (R_a: 1.35 μm), (b) E-CNT1 (R_a: 2.04 μm), (c) E-CNT2 (R_a: 2.43 μm), and (d) E-CNT3 (R_a: 2.53 μm).

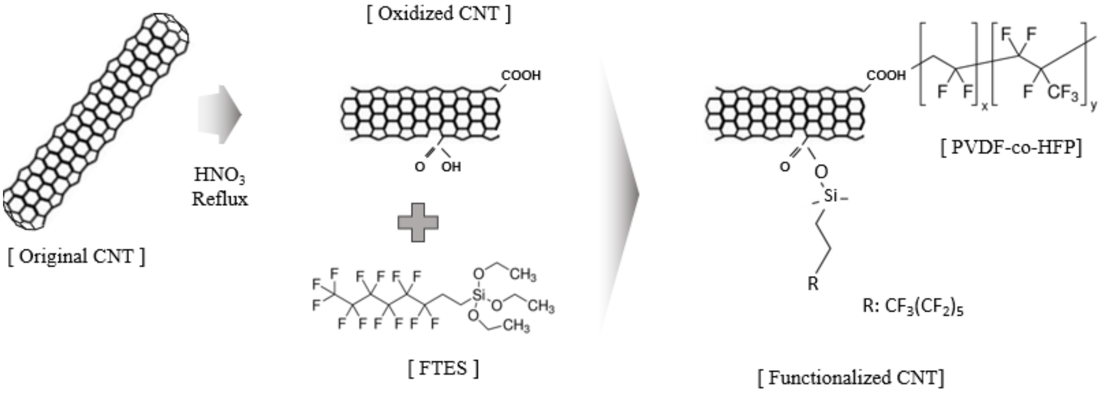

Supplementary Figure S2. A schematic diagram of CNT functionalization steps.

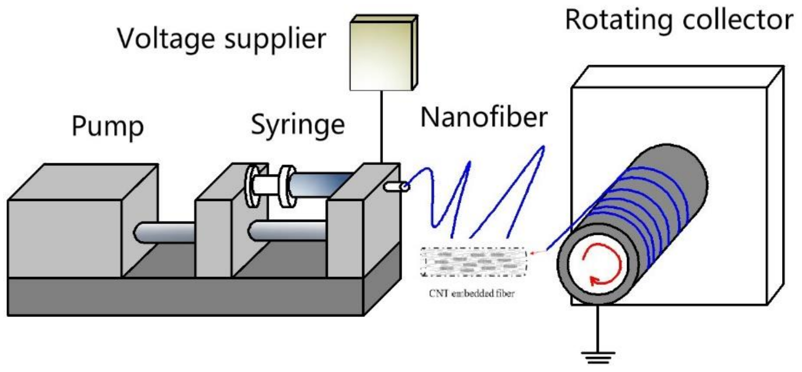

Supplementary Figure S3. A schematic drawing of the electrospinner.

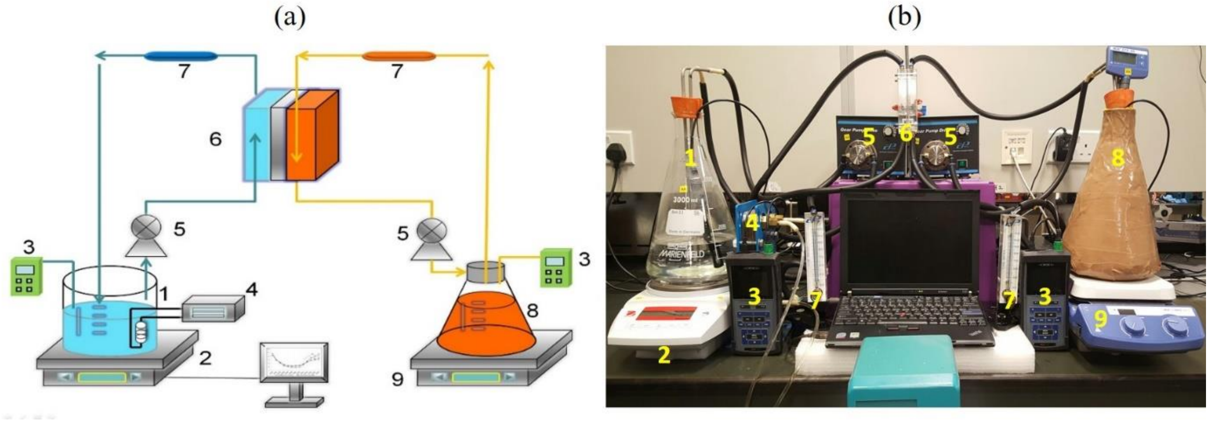

Supplementary Figure S4. (a) A schematic diagram of the DCMD test unit (1: Permeate tank, 2: Digital balance connected to a computer, 3: Thermometers, 4: Cooling unit, 5: Pumps, 6: Flat sheet membrane module, 7: Flow meters, 8: Feed reservoir, and 9: Hotplate). (b) A picture of experimental set-up of the DCMD test unit.

Table S1. Properties and DCMD performance using various types of electrospun membranes. Feed/permeate temperature: 60/20°C. 3.5 wt% NaCl feed solutions.

Material	Mean pore Size (μm)	Porosity (%)	Thickness (μm)	Contact angle (°)	Initial flux ($kg/m^2/h$)	Rejection (%)
[a]67% clay-PVDF [28]	0.64	81.0	300	154.2	5.5	99.95
222% SiO_2-PVDF [33]	0.61	79.7	100	152.3	43.0	99.99
50% TiO_2 -PVDF-HFP [32]	0.75	91.6	100	149.0	40.0	99.99
[b]Fluorinated polytriazole [30]	2.70	-	-	162.0	42.0	99.95
[c]Polystyrene [31]	1.15	77.5	120	150.2	63.2	99.99
E-CNT3 of this study	1.20	89.4	88	150.4	48.5	99.98

[a] Feed/permeate temperature: 80/17°C.
[b] Feed/permeate temperature: 60/22°C. Feed: real sea water.
[c] Feed/permeate temperature difference (ΔT): 55°C.
* Concentration of particles was based on weight of polymer.

Supplementary Table S2. Compositions of dope solutions for the electrospun membranes fabricated in this study.

Electrospun membranes	PH (wt%)	DMF (wt%)	Acetone (wt%)	CNTs (wt% to PH)[(1)]
E-PH	20	64	16	0
E-CNT0.5	20	64	16	0.5
E-CNT1	20	64	16	1
E-CNT2	20	64	16	2
E-CNT3	20	64	16	3

[(1)] Weight percentage (wt%) of CNTs to polymer (PH).
